# The Impact of Pre-analytical Quality Initiatives on Cholangiocarcinoma Diagnostics in Thailand

**DOI:** 10.3389/fpubh.2022.792847

**Published:** 2022-06-10

**Authors:** Supinda Koonmee, Sakkarn Sangkhamanon, Piyapharom Intarawichian, Chaiwat Aphivatanasiri, Waritta Kunprom, Prakasit Sa-ngiamwibool, Suwit Balthaisong, Chitsakul Phuyao, Piya Prajumwongs, Reza Alaghehbandan, Malinee Thanee

**Affiliations:** ^1^Cholangiocarcinoma Screening and Care Program, Khon Kaen University, Khon Kaen, Thailand; ^2^Cholangiocarcinoma Research Institute, Khon Kaen University, Khon Kaen, Thailand; ^3^Department of Pathology, Faculty of Medicine, Khon Kaen University, Khon Kaen, Thailand; ^4^Department of Pathology, Faculty of Medicine, Royal Columbian Hospital, University of British Columbia, Vancouver, BC, Canada

**Keywords:** cholangiocarcinoma, pre-analytical phase, tissue preservation, immunohistochemistry, histopathology

## Abstract

Cholangiocarcinoma (CCA) is the most prevalent malignancy in Thailand, with unfortunate late diagnosis and frequent metastatic disease outcomes. An accurate tissue diagnosis is the first and most important step in the treatment of CCA. Tissue quality and preservation during the pre-analytical phase play major roles in the proper histological evaluation and potential biomarker testing. This study evaluated the impact of using the “Cholangiocarcinoma Screening and Care Program (CASCAP)” container, as an innovative tool to address pre-analytical challenges faced by pathology laboratories in Thailand. This is a comparison study examining the quality of CCA specimens using the CASCAP container vs. the conventional method, using hematoxylin and eosin (H&E) and immunohistochemistry (IHC). CCA tissue quality using the CASCAP container significantly reduced artifact deposition while improving the cellular structure and nuclear and cytoplasmic morphologies. The immunohistochemical expression of cytokeratin 19 (CK19), a prognostic marker in CCA, significantly improved in the CASCAP container group in comparison with the conventional method. This innovation is proven to significantly enhance the CCA tissue quality diagnostics and prognostic biomarker testing, hence improving overall cancer care, diagnosis, and treatment in Thailand.

## Introduction

The Northeastern region of Thailand (also known as Isan), consists of 20 provinces bordered by the Mekong River, which is the capital of cholangiocarcinoma (CCA) with the highest incidence rate worldwide (1). In this region, it has been reported that liver fluke, *Opisthorchis viverrini* (OV), is associated with the high incidence of patients with CCA in Thailand (2–10). Most patients with CCA have a high mortality rate due to a lack of effective treatment and late diagnosis leading to cancer metastasis (11). The current clinical studies with targeted therapies [e.g., monoclonal antibodies and tyrosine kinase inhibitors against epidermal growth factor receptor (EGFR), vascular endothelial growth factor (VEGF), and Fibroblast Growth Factor Receptor 2 (FGFR2)] and immunotherapies (anti-PD-L1) have demonstrated success in terms of overall survival (OS). The association of several biomarkers, such as increased tumor mutational burden (TMB), deficiency in mismatch repair (dMMR) proteins, and high microsatellite instability (MSI-H) predicts immunotherapy drug response in many solid tumors, such as anti-PD-L1 (12, 13). In addition, cancer biomarkers also play a crucial role in guiding adjuvant systemic therapies, cancer recurrence, and potential mechanisms of resistance (14). Hence, the knowledge of CCA molecular pathogenesis with multidisciplinary approaches and advanced technologies has been developed for further CCA treatment. Studies have shown that a panel of protein expression, such as protein kinase can predict the prognosis and metastasis of CCA (15, 16). In addition, available targeted therapies have been described, requiring certain tissue protein expression testing (17–19). Cytokeratin 19 (CK19), a type I cytokeratin, is mostly found in epithelial tissues with high plasticity, such as stem cells, transforming cells, or neoplastic cells. In clinical practice and research, CK19 can be used to differentiate hepatocytes from biliary epithelial cells (20, 21). In CCA, an upregulation of CK19 was positively correlated with aggressive tumor phenotypes and clinical behaviors (lymph node metastasis and larger tumor size) (22, 23). Furthermore, a high expression of CK19 predicts a significantly dismal postoperative survival (23). Importantly, the combined CK7/CK19 index showed to have a prognostic value for survival of patients with CCA, when compared with current tumor staging systems (23). As such, CK19 is an important diagnostic, prognostic, and potential predictive factor in CCA (24).

Immunohistochemistry (IHC) is routinely used in surgical pathology and cancer diagnosis, providing prognostic and predictive information (25–27). IHC is the interaction of an antibody binding to a specific antigen, which is part of a protein. The three-dimensional structure of an antigen is crucial for this interaction. The pre-analytical phase, especially the tissue fixation process, is of paramount importance to prevent protein degradation and structural distortion, potentially leading to reduction in the binding affinity of the primary antibody (28–30).

With the introduction of both precision and personalized medicine, the role of biomarker and molecular testing by pathology laboratories has become more pronounced to provide quality testing (31). The pre-analytical phase is the initial step to preserve tissue quality, such as cold ischemia, fixation, and tissue processing. These factors have been well studied and have become standard practice for breast cancer (32, 33). Hence, in CCA specimen collection, cold ischemic time and fixative duration are not monitored in the conventional method (CV); and, it can introduce challenges in CCA biomarker testing.

In 2015, the senior author (SK) conducted a national project (Pathum Raksa project, which means “Better Breast Cancer Treatment”), as a result of low ER+ breast cancer rates in Thailand, designing a special specimen container through a national innovation to address the issue of poor breast tissue fixation (34). In this study, we adopted the idea of the Pathum Raksa project to examine the impact of utilizing a special specimen container on the quality of CCA diagnostics in northeastern Thailand. We designed a special specimen container (“Cholangiocarcinoma Screening and Care Program (CASCAP)” container), based on the original container design of the Pathum Raksa project (Supplementary Figure S1), to improve pre-analytical processes, such as cold ischemic time, fixative duration, and tissue processing. We compared analytical factors, such as histomorphological and immunohistochemical features between the two methods in CCA cases.

## Materials and Methods

### Human Ethics

The study was approved by the Ethics Committee for Human Research at Khon Kaen University (HE621088).

This is a comparative cross-sectional study assessing histologic and biomarker factors between the current conventional method of tissue handling and using the CASCAP container in CCA diagnostics. Twenty cases of CCA in Srinagarind Hospital, Faculty of Medicine, Khon Kaen University, Thailand, were used in this study, with 10 CCA tissues in each study group.

### CASCAP Container Design

The original container design was developed in 2012 by the senior author (SK) for the Pathum Raksa project (34). In this study, we introduced some modifications to the original design to accommodate for hepatectomy specimens handling. The CASCAP container measures 25 cm × 18 cm × 18 cm, which can contain a maximum of 3 L of formalin when the hepatectomy specimen is placed inside. The container includes four partitions separated by three acrylic plates (25 cm × 18 cm), where each plate has eight pores (2 cm in diameter) evenly distributed (Supplementary Figure S1). The acrylic plates with pores are designed to allow formalin to flow into the tissue in all directions. The hepatectomy specimens are serially sectioned before being placed in the container, allowing optimal formalin exposure. In addition, the acrylic plates provide a surface for fixing and flattening liver tissue, which can prevent tissue rotation and/or distortion. Other advantages of the CASCAP container include easier tissue mapping and sampling as well as facilitating transfer to the pathology laboratory. Moreover, we developed a special surgical requisition form to consistently record key demographic, clinical, and pre-analytical factors. Of note, the container, which differs from routine surgical specimen containers, is easily recognizable when the specimen is transferred to the pathology laboratory.

### Sample Collection, Fixation, and Processing

**Figure 1:**
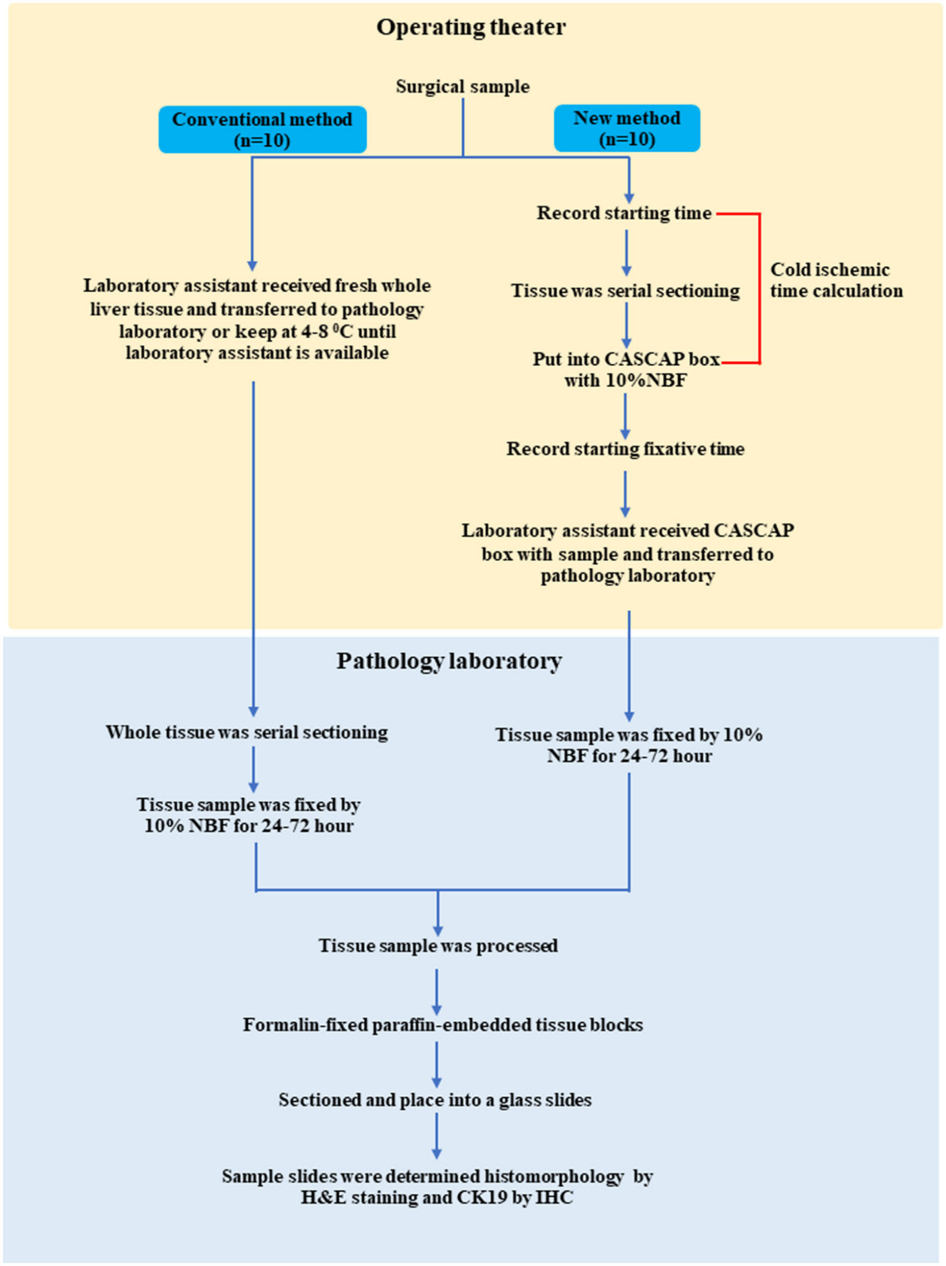
Experimental process.

The study protocol and workflow are shown in Figure 1. In a conventional method, fresh hepatectomy specimens were collected in refrigerator at 4–8°C post-surgery, until the laboratory assistant collected them from operating theater and transferred them to the pathology laboratory. The liver specimens were fixed in 10% neutral buffered formalin (NBF). Cold ischemic time was not recorded, while the fixative duration time was monitored as a standard laboratory procedure within 24–72 h. In this method, several pre-analytical quality factors cannot be monitored, such as cold ischemic time and standard serial sectioning of the specimen to allow proper tissue fixation (Figure 1). In the CASCAP container method, we engaged a multidisciplinary team of surgeons and nurses, who were trained to handle surgical specimens. Briefly, after liver resection, the time was recorded (starting point) on the requisition form by a nurse in an operating theater. Subsequently, the liver specimen was placed in an anatomical position and serially sectioned in ~5 cm in thickness, while ensuring to verify that sectioning passed through the tumor area. The specimen was then placed into the CASCAP container with 10% NBF, and the time of fixation (stopping point) was recorded. The cold ischemic was calculated from the time between the starting point and stopping point. Then, the specimen was transferred to a pathology laboratory and processed within 24–72 h of fixative duration time (Supplementary Figure S1).

Surgical samples in both methods were processed using a similar standard protocol. Formalin-fixed paraffin-embedded tissue (FFPE) blocks were further processed in the Department of Pathology, Khon Kaen University. All paraffin-embedded liver tissues were collected in the specimen bank of the Cholangiocarcinoma Screening and Care Program, the Cholangiocarcinoma Research Institute, Khon Kaen University.

Tissues for light microscopy were embedded in paraffin using a routine procedure. Then, 5 μm thick sections were cut from the tissue blocks and stained with hematoxylin and eosin (H&E). A morphologic scoring was used on H&E stained slides to compare the cellular morphology of the two methods, including areas with pigment artifact, cellular structure, and nuclear and cytoplasm morphology, as described in Table 1.

**Table 1:** Criteria of histomorphology assessment.

**Morphology**	**Scoring criteria**
Formalin pigment artifact deposition (FPAD) (30, 35)	The number of formalin pigment artifact deposition was counted following shown in previous observations.
Cellular structure morphology (36, 37) (Cell shrinkage or Distortion)	Percentage of cellular structure distortion or shrinkage appeared in CCA area
Nuclear morphology (36, 38) (Nuclear detail; Chromatin, Nucleoli Blur,)	Percentage of good nuclear detail Two of three nuclear detail; absent chromatin, absent nucleoli, blur was decided bad nuclear detail in CCA area **Good nuclear detail** **=** Total nucleus - Bad nuclear detail^*^
Cytoplasm morphology (36, 38). (Eosinophilic staining cytoplasm)	Percentage of eosinophilic staining cytoplasm appeared in CCA area

* *Bad nuclear detail was observed and decided*.

### Immunohistochemistry

The immunohistochemical study was performed using a Ventana Benchmark XT automated stainer (Ventana Medical System, Inc., Roche, Switzerland). Immunochemical studies were carried out using CK19 antibodies (Dako, Santa Clara, CA, USA). Appropriate positive and negative controls were used. The CK19 cytoplasmic staining was evaluated using an H-score in 10 fields, based on intensity and percentage of positively stained tumor cells. The intensity was assigned 1–4 scores as follows: 0 (no immunostaining), 1+ (weak intensity), 2+ (moderate intensity), and 3+ (strong intensity). The calculation of H-scores as per earlier studies (39, 40), using the following formula: H-score = [(3 × percentage of strongly staining immunopositive cells) + (2 × percentage of moderately staining immunopositive cells) + (1 × percentage of weakly staining immunopositive cells)], yielded in a range of 0–300 (41). The H-scores were evaluated by two independent pathologists. The median H-score was used as the cut-off value to divide the study groups into low and high CK19 expressions.

### Statistical Analysis

The differences in continuous data between two dependent groups were analyzed by either the independent Student's *t*-test (parametric test) or Mann–Whitney test (non-parametric test). Values were presented as mean ± SD. The chi-square (χ^2^) test was used to examine associations between the fixative methods and the CK19 level. Statistical comparisons between groups were tested using the Student's *t*-test. A value of *p* < 0.05 was considered statistically significant with a two-tail analysis. The data were analyzed by GraphPad Prism® 7.0 software (GraphPad Software, Inc., San Diego, CA, USA) and SPSS 23 software (SPSS, Chicago, IL, USA).

## Results

### Demographic and Clinicopathologic Information

In this study, 20 hepatectomies for CCA were enrolled, with 10 specimens in each group (Table 2). The average patient age was 68 ± 2 years (range, 54–75 years) in the conventional method while it was 67 ± 1.8 years (range, 62–79 years) in the CASCAP method (*p* > 0.05). Patient's gender included 3 women and 17 men. The anatomical location of the tumor included 8 cases of intrahepatic CCA, 8 cases of perihilar CCA, and 4 cases of extrahepatic CCA. The average tumor size in CV was 4.3 ± 0.6 cm (range, 2.5–8 cm.) and 7.2 ± 0.7 cm (range, 3.5–9.5 cm) in CASCAP. The tumor growth pattern showed 10 cases with intraductal growth, 2 cases with periductal infiltrating growth, and 8 cases with mass-forming growth patterns. Histological types included 12 papillary and 8 tubular types. Histological grades included 17 well-differentiated and 3 moderately differentiated. No poorly differentiated CCA was identified. Using the TNM staging [according to the 8th edition of the American Joint Committee on Cancer (AJCC)], patients were staged as pT0 (9 cases; 4 cases of CV and 5 cases of CASCAP), pT2 (3 cases; 2 cases of CV and 1 case of CASCAP), and pT3 (8 cases; 4 cases of CV and 4 cases of CASCAP). Cold ischemic time was not recorded in CV while, the average cold ischemic time in the CASCAP group was 62 ± 5.7 min (range, 45–90 min). No cold ischemic time was recorded as the routine process in the CV group. The fixative duration was monitored within 24 h post-resection in each study group (Table 2).

**Table 2:** Baseline clinicopathological characteristics of the cohort.

**Features**	**Methods**	
	**Conventional (CV)** **(*n* = 10)**	**CASCAP** **(*n* = 10)**
Age (year) (mean ± SEM)^*^	68 ± 2 (range, 54–75)	67 ± 1.8 (range, 62–79)
**Sex**		
Female	1	2
Male	9	8
**Anatomical location**		
Intrahepatic CCA	3	5
Perihilar CCA	6	2
Extrahepatic CCA	1	3
Tumor size (cm) (mean ± SEM)^*^	4.3 ± 0.6 (range, 2.5–8)	7.2 ± 0.7 (range, 3.5–9.5)
**Growth pattern**		
Intraductal growth	6	4
Periductal infiltrating growth	2	0
Mass forming growth	2	6
**Histological types**		
Papillary	5	7
Tubular	5	3
**Histological grade**		
Well	9	8
Moderate	1	2
Poorly	0	0
TNM stage by 8th edition AJCC^*^staging system		
Stage 0	4	5
Stage I	0	0
Stage II	2	1
Stage III	4	4
Stage IV	0	0
Cold ischemic time (minute), (mean ± SEM)^**^	-	62 ± 5.7 (range, 45–90 min)
Fixative duration (hour)^***^	24	24

* *Eighth edition The American Joint Committee on Cancer*.

** *Mean ± SEM was used in this study*.

*** *Fixative duration was regulated at 24 h in two methods*.

### Histologic Parameters

**Figure 2:**
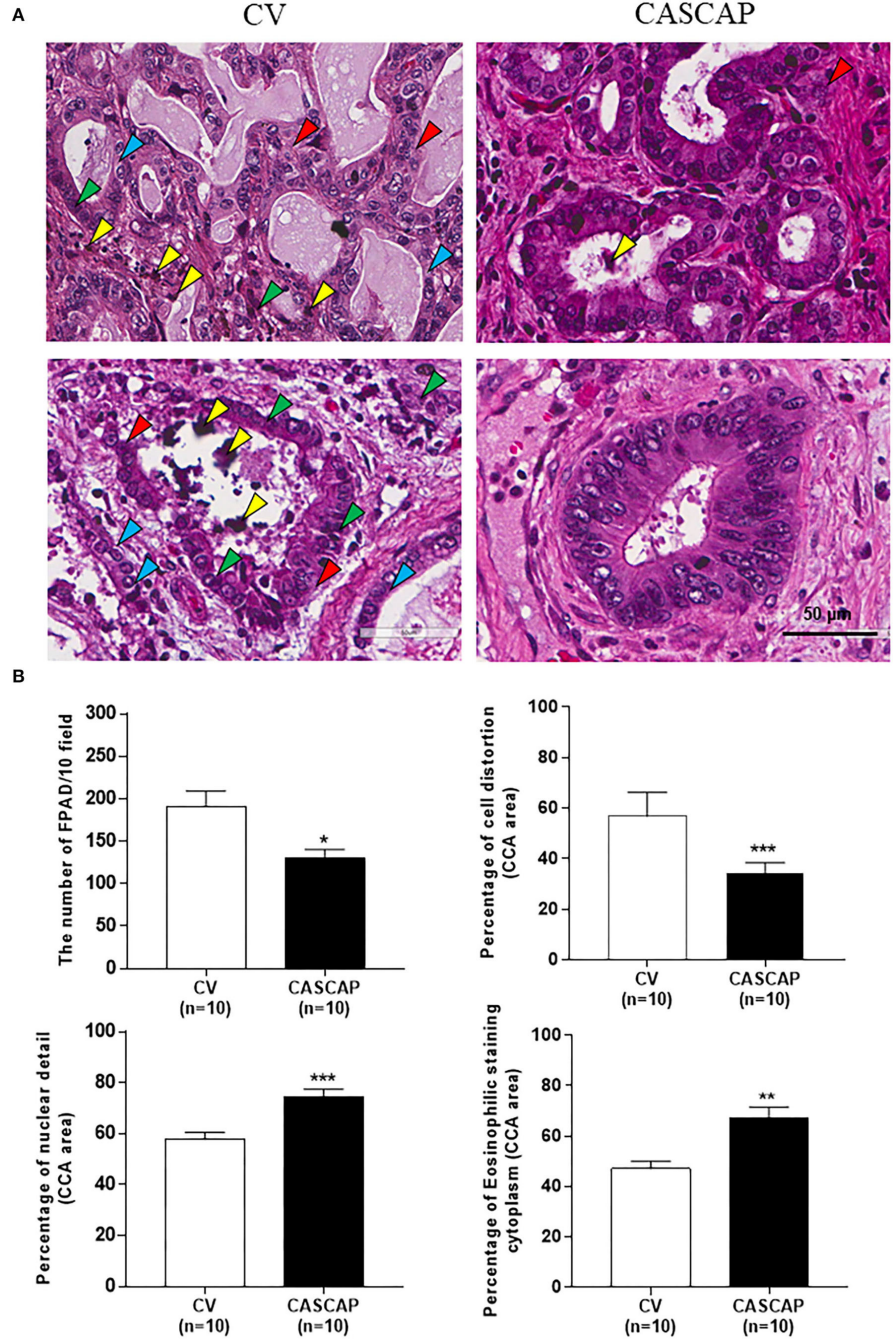
Histopathology of cholangiocarcinoma (CCA) tissues using H&E staining by the two methods, conventional (CV) and new [Cholangiocarcinoma Screening and Care Program (CASCAP)] methods. A comparison of CCA tissue morphologies with staining and structural quality between CV and CASCAP methods. Yellow arrow; formalin pigment artifact deposition (FPAD); Red: cellular structure; green and blue: nuclear and cytoplasm morphologies **(A)**. The numbers of FPAD in 10 fields/slide and the percentage of each phenotype, such as structural distortion, nuclear details, and eosinophilic staining cytoplasm were determined and shown as mean ± SEM **(B)**. ^*^p < 0.05, ^**^p < 0.01, and ^***^p < 0.001.

The following histological features, such as formalin pigment artifact deposition (FPAD), cellular structure, and nuclear and cytoplasmic morphologies, were assessed between the two groups using the criteria presented in Table 1. FPAD was significantly decreased in the CASCAP group in comparison with the conventional method (*p* < 0.01). Cellular changes, such as shrinkage and distortion were remarkably improved in the new method while the conventional method had cellular shrinkage and distortion (*p* < 0.001). In addition, the number of nuclear details, such as nucleoli and mitoses, was much more preserved in the CASCAP method compared with the conventional method (*p* < 0.001). Moreover, the loss of eosinophilic staining in the cytoplasm in the conventional method was significantly higher than in the new method as shown in Figures 2A,B (*p* < 0.01).

### CK19 Immunohistochemical Staining

**Figure 3:**
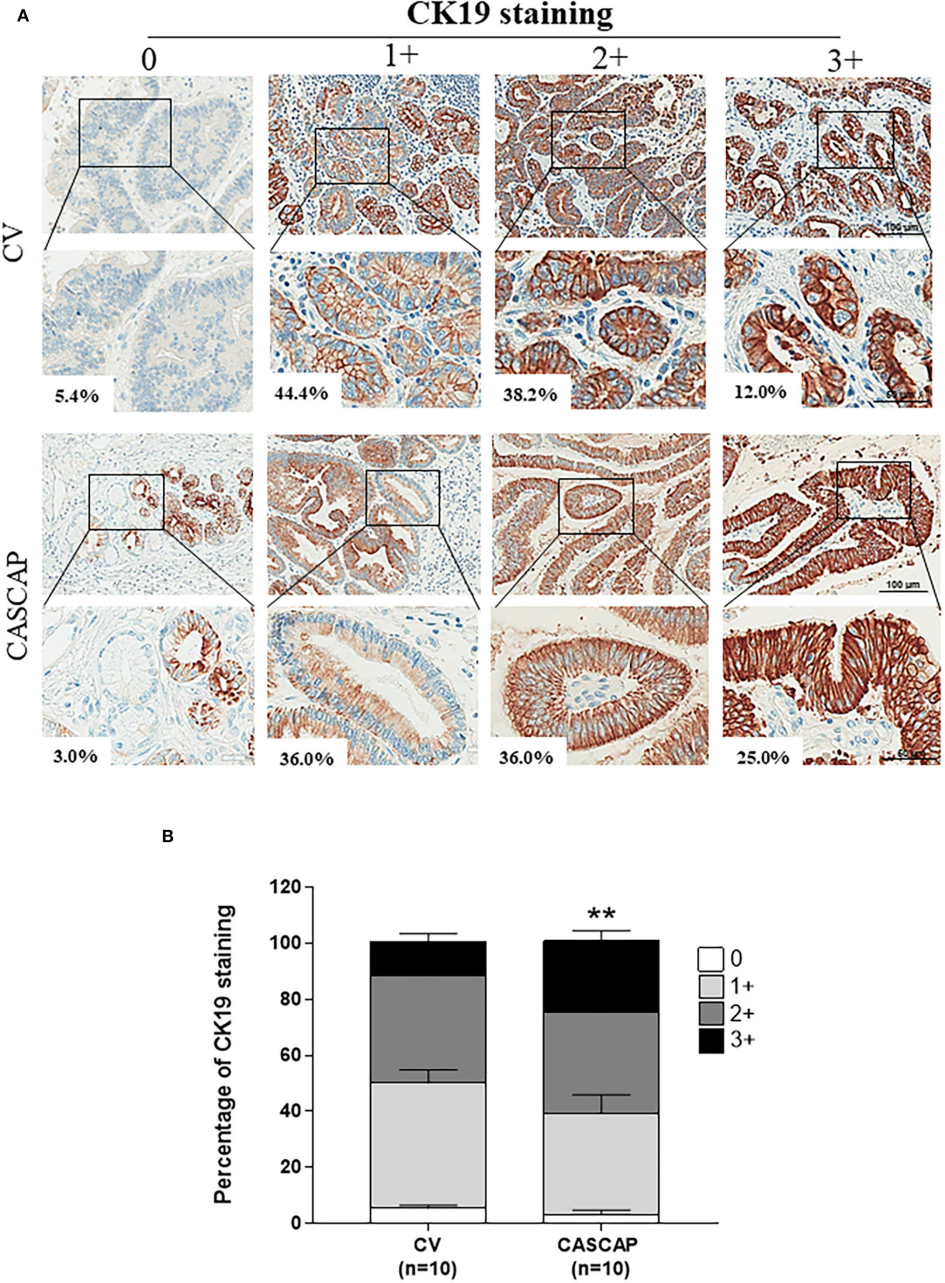
Immunohistochemical assessment of cytoplasmic CK19 intensities in CCA tissue sections from 0 to +3 **(A)**. Percentages of each cytokeratin 19 (CK19) intensities between conventional method (CV) and new methods (CASCAP) **(B)**. Data are shown as mean ± SEM, **p < 0.01.

The neoplastic cells in the CASCAP group showed a higher percentage of CK19 staining level (as much as 25%), while in the conventional method this was only shown at 12% (*p* < 0.01) (Figures 3A,B).

**Figure 4:**
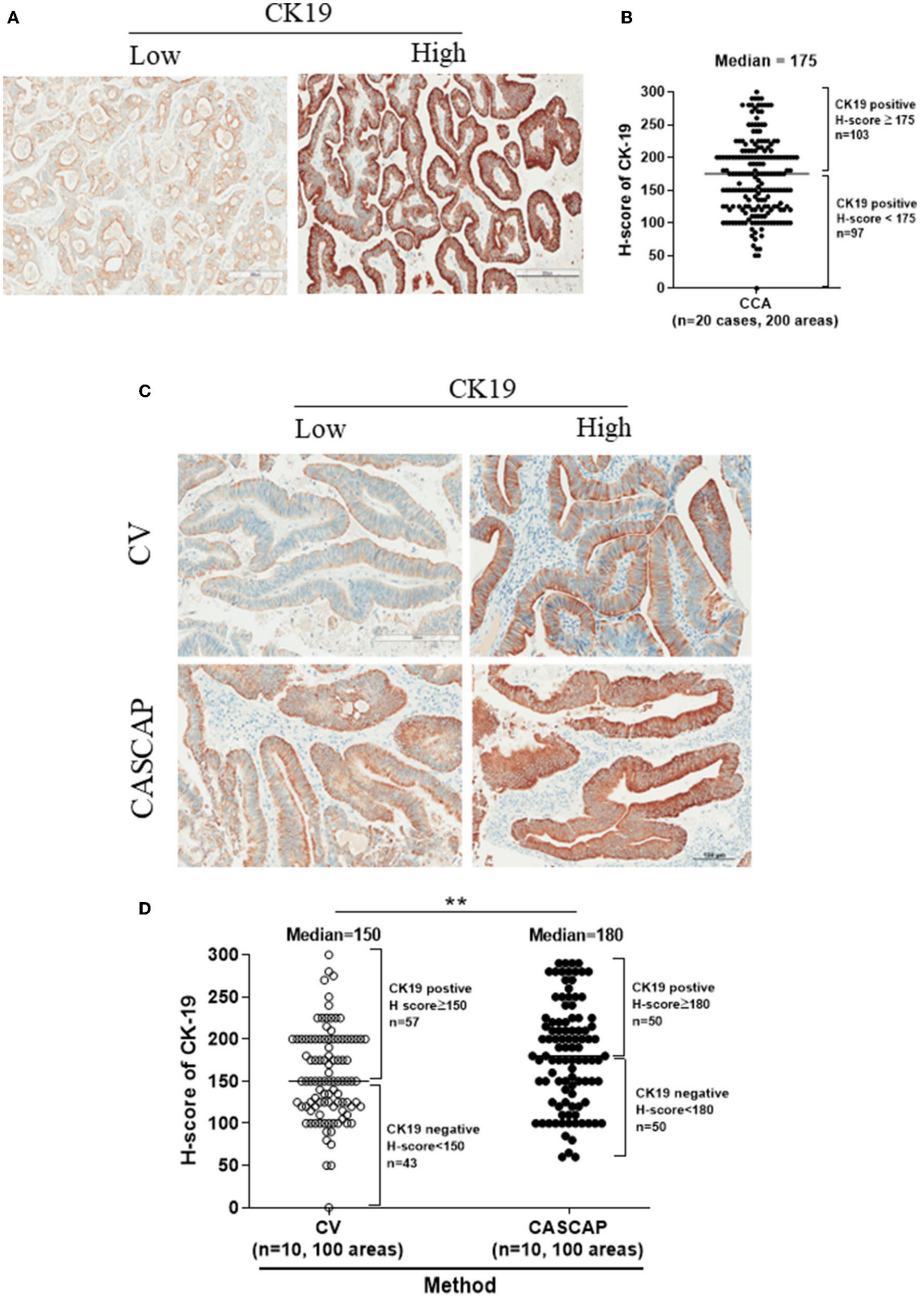
The expression levels of CK19 in total cases were categorized by the median of H-score (175) including low (H-score < 175) and high CK19 (H-score ≥ 175) **(A,B)**. The individual category of CK19 levels in each method **(C,D)** were present as mean ± SEM. **p < 0.01.

Figures 4A,B showed the expression of CK19 in total cases that were separated by the median of H-score including low (H-score < 175) and high (H-score > =175) CK19. Our findings showed that the new method was significantly associated with a higher level of CK19 expression, while the conventional method was associated with a lower level (p < 0.01) (Table 3). In addition, our results showed the CK19 levels in the new method (181.9 ± 6.17) were significantly higher than that seen in the conventional method (156.7 ± 5.33) (p < 0.001) (Figures 4C,D; Table 4).

**Table 3:** Association of CK19 staining between new (CASCAP) and conventional (CV) methods.

**Method**	**CK19 level**		**Total**	***P*-value**
	**Low (H-score < 175)**	**High(H-score ≥ 175)**		
Conventional	58	42	100	0.007
CASCAP	39	61	100	
Total	97	103	200	

**Table 4:** Comparison of CK19 levels between new (CASCAP) and conventional (CV) methods.

**Method**	**CK-19 level**			
	**Mean ±SEM**	**Median (cut-off value)**		
			**Low (*n*)**	**High (*n*)**
Conventional	156.7 ± 5.33	150	43	57
CASCAP	181.9 ± 6.17^**^	180	50	50

** *p < 0.001*.

**Figure 5:**
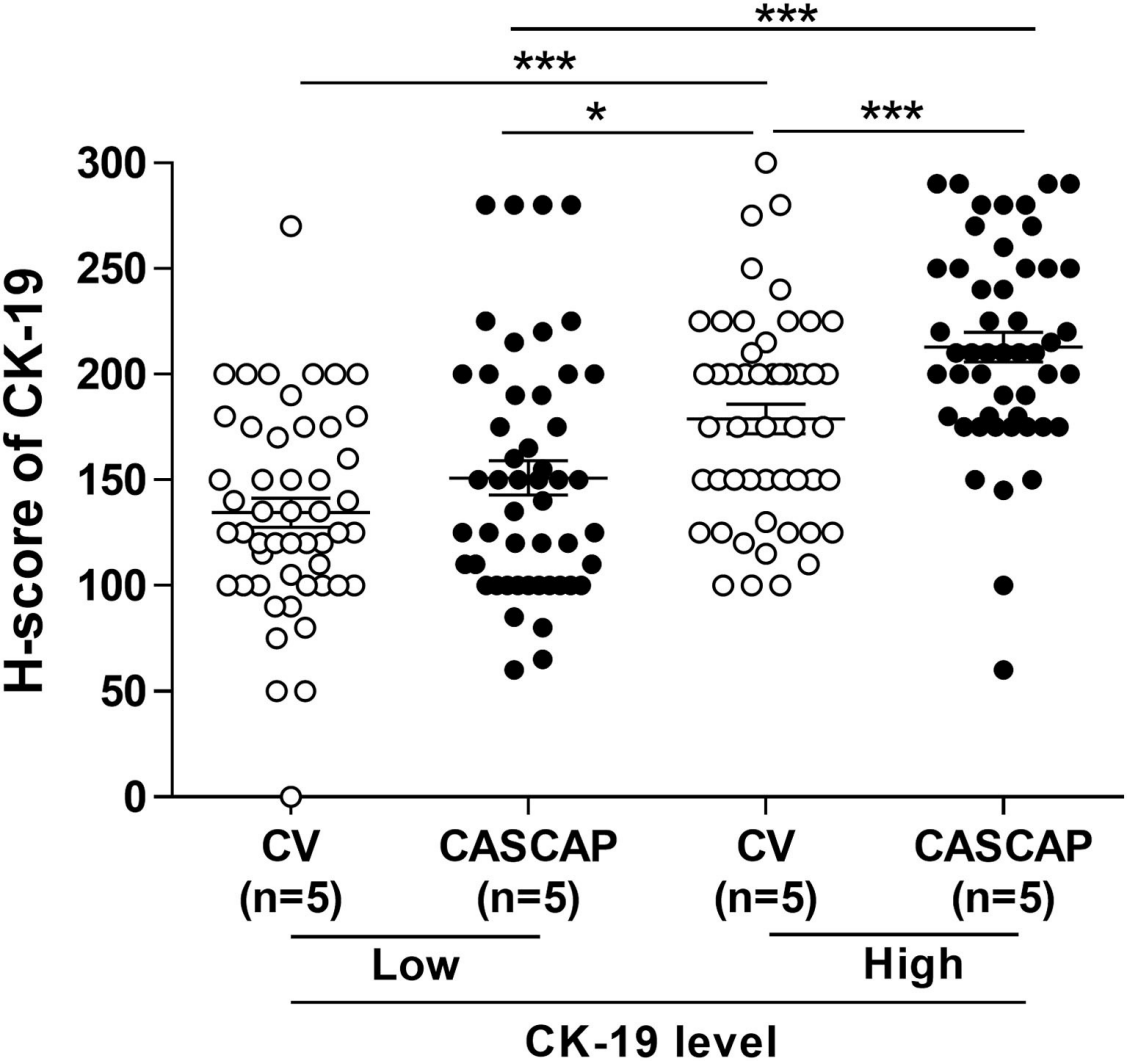
Comparison of low and high CK19 signals between conventional (CV) and new (CASCAP) methods was demonstrated as dot plot. CASCAP, H-score < 180 as low and H-score ≥ 180 as high and CV, H-score < 150 as low and H-score ≥ 150 as high. *p < 0.05 and ***p < 0.001.

To validate that CK19 staining in the new method was increased, the median CK19 score was used as the cut-off value to divide the group into low and high. The CK19 values were 150 for conventional and 180 for the CASCAP group (Table 5). Interestingly, the mean CK19 signal in the CASCAP method (212.9 ± 6.96) was significantly higher than that of the conventional method (178.9 ± 6.95) (p < 0.001). Even in the low CK19 group, the CK19 value in the CASCAP group (150.9 ± 8.13) was higher than the conventional cohort (134.5 ± 6.81) (p = 0.125) (Figure 5; Table 5). Significantly, the gap between low and high CK19 expressions in the new method (62) trended to be higher than in the conventional method (44.4).

**Table 5:** Comparison of CK19 level and gap between subgroup of new (CASCAP) and conventional (CV) methods.

**Method**	**CK-19 level (mean ± SEM)**		**Average**	**Gap (high-low)**
	**Low**	**High**		
Conventional	134.5 ± 6.81	178.9 ± 6.95	156.7 ± 5.33	≈44.4
CASCAP	150.9 ± 8.13	212.9 ± 6.96	181.9 ± 6.17	≈62
Average	142.7 ± 5.33	195.9 ± 5.18		

## Discussion

In this study, we utilized an innovative method to improve the quality of CCA diagnostics in Thailand. One of the most important analytical factors in cancer diagnostics is the pre-analytic phase. The pre-analytical phase consists of cold ischemia, fixation, and tissue processing to maintain tissue quality for histologic diagnosis and potential biomarker testing. One of the immediate and unique features of this project was to engage the surgical team including the surgeons and operating room (OR) staff in the pre-analytical process, where they are not routinely involved. A multidisciplinary team of surgery and pathology departments led to address the issue of cold ischemic time and tissue fixation by monitoring the ischemic time, serially sectioning the specimen, and using a CASCAP container. Of note, none of these would have taken place in a routine conventional method of handling specimens. The issue of cold ischemic time and proper fixation duration has well been studied, particularly in breast cancer biomarkers (32, 33, 42). Formalin fixation time is an important factor in the pre-analytic phase impacting the quality of RNA and miRNA-seq profile and ultimately biomarker testing (38, 43–45). Similarly, studies have shown that the pre-analytical phase impacts the quality of cell morphology, proteins, and nucleic acids (36, 38, 44, 46, 47).

In the pre-analytical phase, adverse tissue changes will mostly be impacted by prolonged cold ischemic time. In this study, cold ischemic time was recorded in the operating theater for the CSACAP group, while in the CV cohort, no monitoring as per routine was documented. The recommended and ideal cold ischemic time has been suggested to be <60 min to preserve biomarkers (48–50). In this study, the cold ischemic time was ~45–60 min. Further, hepatectomy specimens were serially sectioned (~5 cm in thickness) and collected in the CASCAP container with a 10% NBF fixative solution. Considering that the formalin diffusion rate is 1 mm/h in all directions, the tissue fixation would have been completed within 24 h (51). On the other hand, in the conventional method, the specimens were placed in a routine container with no serial sectioning in the operating theater and transferred to a pathology labolatory. Our findings showed that CCA tissue samples were processed and fixed well in the new method with the CASCAP container, reducing tissue damage by autolysis of proteins and preserving cellular structures, which is consistent with the findings of other studies (28–30, 38, 44, 46).

The treatment of CCA is evolving with the introduction of novel biomarkers (52). In addition, targeted therapies may play a crucial role in CCA treatment, such as EGFR (19). Many studies showed that the IHC expression of several proteins in CCA can vary depending on the severity of the disease and correlate with prognosis. Evaluation of a panel of kinase proteins in CCA tissues using IHC demonstrated high expression of EGFR, HER4, and EphA3 to be associated with recurrence-free survival (RFS) and OS (16). Moreover, the increased expression of cancer stem cell markers, such as CD44 and its variants (CD44v6 and CD44v8-10), was also correlated with short RFS and poor prognosis (15). In addition, CK19 was found to be related to CCA metastasis and poor survival (23, 24, 53). Since IHC is routinely used in pathology diagnosis (25–27) and may play a crucial role in precision medicine, preservation of antigen quality is necessary to support clinically indicated target therapies. Although several studies have demonstrated the expression of various proteins in CCA, the study of pre-analytic analysis, sample preparation, and preservation has not well been explored. Preservation of the specimen during the pre-analytical phase is crucial to ensure tissue quality, which ultimately impacts the analytical phase. Thus, this study is the first to provide evidence and demonstrate that our proposed methodology is significantly superior in preserving the tissue antigenicity, hence improving IHC interpretation.

In this study, the intensity of CK19 immunostain in the new method was significantly higher than that seen in the conventional method. Other studies have also suggested that the quality of tissue fixation impact antigenicity and provides binding affinity between antigen-antibody interactions (28–30, 54, 55). Our innovation showed that the pre-analytical phase can improve the preservation of tissue quality by addressing two major quality factors: (1) monitoring and optimizing cold ischemic time via a multidisciplinary team, and (2) increasing fixative diffusion in the tissue sample due to partition plates with pores in the CASCAP container. We are hoping to extend and introduce this innovation to the rest of the country to improve cancer diagnostics and care in patients with CCA.

Through an innovative initiative, a CASCAP container was designed to optimize the pre-analytical quality factors. This was also conducted by a multidisiciplinary team of surgery and pathology departments. The innovative method significantly improved tissue preservation and immunohistochemical detectability of CK19 staining. This can also be utilized for detecting other proteins that might be used as potential biomarkers for predicting CCA prognosis, recurrence as well as chemotherapeutic drug response. We hope and intend to extend and introduce this innovation to the rest of the country to improve cancer diagnostics and care in patients with CCA.

Although the newly established CASCAP procedure is better than the conventional procedure, less cellular changes, more nuclear details, and higher positive rate for CK19. This study is pilot study in which the Pathum Raksa Project in breast cancer concept was applied to cholangiocarcinoma. This study faces limitations including rather small sample size. This study investigated the impact of CASCAP on only one antigen. A larger number of antigens detection would be necessary to investigate and validate the protein quality. In addition, this study demonstrated only protein sample. Other samples, such as the DNA or RNA samples are need to validate preservation ability of CASCAP.

## Data Availability Statement

The original contributions presented in the study are included in the article/Supplementary Material, further inquiries can be directed to the corresponding author/s.

## Ethics Statement

The studies involving human participants were reviewed and approved by Ethics Committee for Human Research, Khon Kaen University. The patients/participants provided their written informed consent to participate in this study.

## Author Contributions

Conceptualization, funding acquisition, and supervision: SK. Formal analysis: SK, MT, SS, PI, CA, WK, and PP. Investigation: SB and CP. Writing—original draft: MT and PP. Writing—review and editing: SK, SS, PI, CA, RA, and WK. All authors approved the final version of the manuscript.

## Funding

We would like to acknowledge the Cholangiocarcinoma Screening and Care Program (CASCAP) under Cholangiocarcinoma Research Institute (CARI), Khon Kaen University, Khon Kaen, Thailand, and The National Research Council of Thailand through Fluke Free Thailand project, Khon Kaen University through Cholangiocarcinoma Research Institute to support funding and the patient information and tissue samples and Department of Surgery, Faculty of Medicine, Khon Kaen University, Khon Kaen, Thailand for assistance sample resection and handling following our methods.

## Conflict of Interest

The authors declare that the research was conducted in the absence of any commercial or financial relationships that could be construed as a potential conflict of interest.

## Publisher's Note

All claims expressed in this article are solely those of the authors and do not necessarily represent those of their affiliated organizations, or those of the publisher, the editors and the reviewers. Any product that may be evaluated in this article, or claim that may be made by its manufacturer, is not guaranteed or endorsed by the publisher.
