# Supplementary material for: The Impact of Pre-analytical Quality Initiatives on Cholangiocarcinoma Diagnostics in Thailand
Source: Front Public Health. 2022 Jun 10;10:792847. doi: 10.3389/fpubh.2022.792847 (PMC9231639; doi:10.3389/fpubh.2022.792847)
Supplement: Supplementary file 1 [file Image_1.pdf]

Supplementary Material

Supplementary Figures

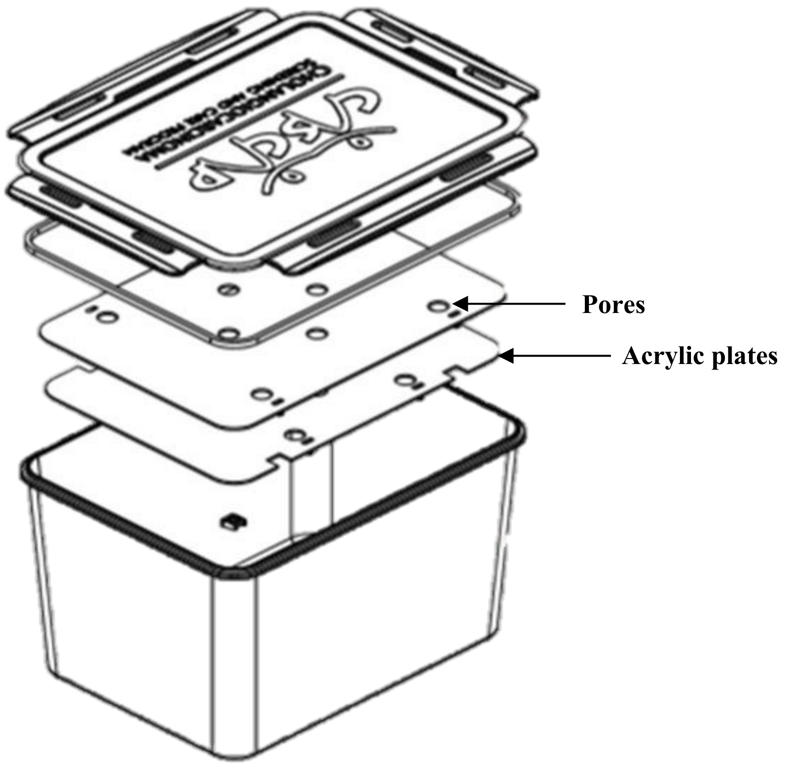

Supplementary Figure 1. The CAPCAP box structure contained three Acrylic plates with pores that separated each floor of the box. It was suitable to contain liver tissue.
